# Social Outcomes of School Leavers With Cerebral Palsy Living in Victoria

**DOI:** 10.3389/fneur.2021.753921

**Published:** 2021-12-14

**Authors:** Christine Imms, Dinah Reddihough, Daisy A. Shepherd, Anne Kavanagh

**Affiliations:** ^1^Apex Australia Foundation Chair of Neurodevelopment and Disability, Department of Paediatrics, The University of Melbourne, Parkville, VIC, Australia; ^2^Principal Research Fellow, Murdoch Children's Research Institute, Melbourne, VIC, Australia; ^3^Biostatistician, Department of Paediatrics, The University of Melbourne, Parkville, VIC, Australia; ^4^Chair in Disability and Health, School of Population and Global Health, The University of Melbourne, Parkville, VIC, Australia

**Keywords:** economic participation, social outcomes, life satisfaction, survey method research, cerebral palsy, young adult

## Abstract

**Objective:** In Australia, the National Disability Strategy provides a framework to guide actions and investment to achieve equity in social inclusion and economic participation for people with disability. We investigated the social outcomes of school leavers with cerebral palsy (CP) in Victoria, Australia and explored the determinants of desirable outcomes.

**Methods:** We used the Victorian CP Register to invite all adults with CP aged 18–25 years (*n* = 649). On-line and/or paper-based surveys explored participation in education, employment, community activities, living situation, relationships and life satisfaction. Functional and health status data were collected. Social outcomes were summarized descriptively and compared between individuals with CP and non-disabled peers aged 18–25 years from the Household Income and Labor Dynamics in Australia dataset. Within the CP cohort we explored whether physical and mental health and level of functioning were associated with social outcomes. In addition, a descriptive comparison was undertaken between the social outcomes of the current CP cohort with that of a previously reported 2007 cohort.

**Results:** Ninety participants (57% male; mean age 22.4 years (SD: 2.2) in 2020; 61.1% self-reported) provided data for analyses; response rate 16.9%. CP characteristics were similar between respondents and non-respondents. In comparison to similar aged peers, 79.8% had completed secondary school (compared to 83.2%); 32.6% (compared to 75.8%) were in paid work; 87.5% (compared to 48.2%) were living in their parental home; and 3.4% (compared to 31.6%) were married or partnered. Individuals with CP and higher levels of functional capacity and better physical health were more likely to undertake post-secondary education. Higher levels of functional capacity and physical health, as well as lower mental health status were associated with being employed.

**Conclusions:** While foundational education completion rates were similar to non-disabled peers, significant gaps in social outcomes remain, including residence in the parental home and single status. While addressing these issues is challenging, substantial efforts are needed to reduce these disparities—work that needs to be done in collaboration with people with CP and their families.

## Introduction

Adults with cerebral palsy (CP) experience social and economic inequalities and challenges (1–3), related to impairments associated with CP (4), societal barriers such as discrimination, and a lack of accommodations they require to cater for their needs (5, 6). There is evidence that participation in life situations becomes more challenging as children with CP transition to adulthood. As their social roles change, they expect and desire to participate in common adult activities such as employment, further education, independent living and intimate relationships (7).

CP is a lifelong condition that occurs as a result of damage to the developing brain in utero or in the first 2 years of life (8). While the primary disorder is of movement and posture, people with CP experience a range of co-morbidities (e.g., intellectual impairment, epilepsy, hearing or vision impairment, musculo-skeletal contractures and deformities) and functional impacts (8). For example, almost half require assistance to mobilize, two-thirds require help to handle objects, one-fifth require assistance to communicate, one quarter have behavioral concerns and three-quarters experience chronic pain (9). While prognostic data is most commonly found in studies of children, more recent research that has focused on adults, confirms the epidemiology of ongoing functional impacts of CP into adulthood (10). Given that in Australia over 75% of the estimated 35,500 individuals who have CP are adults (11), evidence of outcomes for Australian adults with CP is needed.

The 2010–2020 National Disability Strategy provided a policy framework to guide investment and activity to improve performance of mainstream services for people with disability, increase the visibility of disability issues and improve inclusion of those with disability (12). The second 10-year National Disability Strategy will be released late 2021. In 2013, within the life of the 2010–2020 Strategy, a National Disability Insurance Scheme (NDIS) was legislated for implementation, representing a major policy shift and re-direction of disability funding (13). The intent of the NDIS was to give choice and control to individuals with severe and permanent disability *via* funding packages which they could use to purchase supports and services (14). The NDIS funding is explicitly to support individuals to meet their goals for participation in the usual activities of life (13) (see also, www.ndis.gov.au). In 2018, ~5% of NDIS recipients were those with CP, however, they required the second highest annualized support package in dollar value (11), emphasizing that an understanding of participation gaps and supports required, is needed.

In 2007, we investigated the social situation of 335 young adults with CP aged 20–30 years (2). Our results showed that compared to their non-disabled peers, many of those with CP had not completed secondary education (50 vs. 20%), were unemployed (64 vs. 20%), and had an annual income of < $20,000 Australian dollars (80 vs. 39%). These findings have been substantiated in more recent international research. For example, a series of studies from The Netherlands (7), found that difficulties in participation in domestic life and interpersonal relationships for individuals with CP increased from the age of 16 years. In related work, van Wely et al. (15) demonstrated that family and daily supports, along with personal coping styles and prior participation patterns predicted participation in adulthood in domestic life and interpersonal relationships. Schmidt et al. (16) also found that individuals with CP appeared to lag behind their age-matched non-disabled peers in the development of autonomy in life domains, including finances, intimate relationships and housing. Those with lower gross motor function were at risk of not achieving full autonomy. This body of work from The Netherlands provides important evidence but is limited by its exclusion of individuals with CP with intellectual impairments. In Sweden, Jacobson et al. (17) also described the social participation of young adults with CP aged 20–22 years, including those with intellectual impairment. Jacobson et al. (17) found young adults with CP were likely to be living in the parental home, be unemployed and dependent on family for financial and basic support for daily activities. Comparisons with age-matched peers were not provided in that study, but the relationships between communication, intellectual and manual abilities and participation outcomes was highlighted (i.e., higher levels of activity limitation were associated with decreased participation).

Research that involves adults with CP has been growing rapidly over the past two decades. A recent overview of systematic reviews of research about adults with CP identified 19 reviews and mapped their content and focus to the domains of the International Classification of Functioning, Disability and Health (ICF) (18). One finding of the overview was that the predominant focus of research has been at the ICF level of body functions, with 12 of the 19 included reviews mapped to this aspect (19). While an overview of systematic reviews does not identify all pertinent research, nevertheless it does highlight the need to shift the focus in research with adults with CP beyond mobility and gait, to broader social outcomes. The need for a change in focus is supported by Lindsay's (20) qualitative synthesis—a systematic review of lived experiences of children and youth with CP. Lindsay found that young people's accounts of their experience of CP focused on social inclusion and aspects of the environment (i.e., family, peers, services, supports) and when aspects of body functions were discussed, they were more likely to be pain, fatigue and mental health, rather than mobility, assistive devices and activities of daily living.

Ensuring optimal outcomes for young people with CP is crucial so that they can participate fully in home and community life. We have little knowledge of how Australian young adults with CP are progressing at the current time and what could be put in place to improve their social and economic participation. When young people are at school, they often receive considerable support and are linked with a range of services. When they leave school, there are significant changes in their care and management (21). Some attend higher education facilities, others seek open or supported employment, whilst others may participate in community activities or in day centre programs. With the advent of the NDIS and the National Disability Strategy in Australia, it is crucial that information is generated so that gaps in service provision can be addressed and that the needs of the individuals themselves, and their families, can play a central role in guiding allocation of resources into the future.

The purpose of this study, therefore, was to investigate the social outcomes of young adults with CP (aged 18–25 years) in Victoria, Australia and determine the factors that may result in desirable outcomes. While this cohort was not fully comparable [included age range of the previous study was 20–30 years, mean = 24.7 years (SD 2.8)] to the 2007 cohort (2), we also aimed to explore whether social outcomes had changed since the study 13 years ago. The specific research questions were:

1. What are the social outcomes of Victorian young adults with CP in terms of further education, employment, living situation and community participation?

a. How do educational, employment, living arrangements, marital status and financial outcomes compare to young adults without CP?
b. Are the social outcomes of the current cohort comparable to that of the 2007 cohort of young adults with CP living in Victoria, Australia?

2. What are the determinants of social outcomes for young adults with CP aged 18–25 years in terms of individual (e.g., functional or health status) and environmental (e.g., socio-economic status, educational background) factors?
3. How satisfied are young adults with CP, or those responsible for their care, with their outcomes?

## Methods

### Design

This descriptive study used survey methods and secondary analyses of matched datasets to address the research aims. Our multi-disciplinary research team also engaged with stakeholders (young adults with CP and parents) as a consultation group during inception of the study.

### Ethical Considerations

Ethical approval for the study was received from the Australian Catholic University Human Ethics Committee (ACU HREC 2018–43H). Participants provided written informed consent to participate in the study, with substitute decision makers (e.g., parent, primary carer) providing consent on the behalf of participants who did not have the capacity to consent. As we aimed to recruit participants with a diverse range of impairments, options for survey completion were provided as follows: (i) choices made by myself and filled out by myself; (ii) choices made by myself and filled out by another person; or (iii) choices made on my behalf and filled out by another person.

### Participants and Recruitment

Participants were eligible to participate in the study if they were aged 18–25 years, had a diagnosis of CP of any type or severity, no longer attended secondary school and currently lived in Victoria, Australia. Participants were also included *via* a proxy respondent (parent, or responsible other identified by the individual) if they were unable to complete the survey on their own. Efforts were made to engage and include individuals across all five Gross Motor Function Classification System (GMFCS) (22) levels and those with difficulties relating to communication and/or intellectual function to achieve diversity of participants' experience.

Participants were recruited for the study *via* letters of invitation through the Victorian Cerebral Palsy Register (VCPR; *n* = 649 eligible), advertising in newsletters of the Cerebral Palsy Support Network and snowball invitations between participants and social media posts. Follow up letters were sent to those with current addresses on the VCPR as reminders.

### Data Collection

The survey questions were developed following a literature review to explore participation of school leavers with CP. The review, plus consultations with a reference group of adults with CP, was used to identify the important life situations to include as well as potential factors to consider when designing the survey. The result was a survey that sought information about (i) the following life situations—further education, employment, friendships and intimate relationships, living situation, health care provision, community participation; (ii) individual factors influencing participation—CP functional status, mental and physical health status, personal factors (gender, education attainment); (iii) environmental factors influencing participation (parental roles and education, school setting, transport availability, provision of health services); and (iv) life satisfaction. We then sought valid and reliable outcomes to measure the identified variables (see Table 1) to which items about participation in education, employment, living situation and community life were added, to create a compilation of measures as an online or paper-based survey. The survey comprised 89 items and took ~30 mins to complete.

**Table 1:** Validated measures included in the compiled survey.

**Variable**	**Measure/s**	**Validity/reliability**	**Scores/interpretation**
**Cerebral palsy descriptors of functional levels**			
Gross motor function	GMFCS E & R (22)	Inter-rater reliability therapists/parents Kappa = 0.716 (CI 0.596 to 0.836)	Ordinal−5 levels; I to V with V = no independent mobility
Manual ability	MACS (23)	Rater reliability parents/therapist ICC 0.96 (0.89–0.98)	Ordinal−5 levels; I to V with V = severely limited manual ability
Communication function	CFCS (24)	Test–retest reliability 0.82.	Ordinal; 5 levels; I to V with V = seldom effective sending or receiving
Eating and drinking ability	EDACS (25)	Rater reliability parent/ professional κ = 0.82	Ordinal−5 levels; I to V with V = unable to eat and drink safely
**Mental and physical health**			
Global health	PROMIS Scale V1.25 Global health (26)	Good internal consistency for two scales (α = 0.82–0.88)	Scale—High scores better health; Score Mean = 50, SD 10 Item scores: 1–5
Mental health	K10 measure of anxiety and depression (27)	Good internal consistency: α = 0.93	Scale—High scores worse mental health: <20: likely well 20–24 mild 25–29: moderate 30+: severe disorder
**Measures of variables that may influence participation in life situations**			
Psychosocial job quality	Scales from HILDA survey (28) Job demands and complexity Job security Job control	Factor structure and validity confirmed Internal consistency range: α = 0.60 to 0.82	Ordinal—High scores = higher job adversity (worse); Range: 0 to 6
Availability of supports	RAND social support scales: Emotional/informational Tangible Affectionate Positive social interactions (29)	Factor structure and validity confirmed Internal consistency of scales range: α = 0.91 to 0.96	Scale—High scores = more available support; Range: 0–100
**Life satisfaction**			
Wellbeing/ satisfaction	Personal Wellbeing Index (30) 8 items: Life as whole Standard of living Health Life achievements Relationships Feeling of safety Part of community Spirituality	Factor structure, validity and reliability established; Test-retest reliability: ICC = 0.84	High scores = high satisfaction Range: 0–10 scales Australian normative range (scale converted 0–100): 73.4–76.4

*GMFCS, Gross Motor Function Classification System; MACS, Manual Ability Classification System; CFCS, Communication Function Classification System; EDACS, Eating and Drinking Ability Classification System; HILDA, Household Income and Labor Dynamics in Australia; α, alpha coefficient; ICC, intra-class correlation coefficient*.

Demographic data were also collected, including gender, date of birth, Indigenous status, parents' education, employment and place of birth, and languages spoken at home. The resultant survey was reviewed and piloted to ensure clarity of understanding with a small number of individuals known to the researchers, and modifications were made as necessary. While the survey was intended to be delivered in an online environment, for respondents who could not, or preferred not to access the online version, a paper-based version of the survey was also available. The online survey was supported and distributed using the web-based application Research Electronic Data Capture (REDCap) (31, 32). Data collection occurred during the latter part of 2019 and ceased in March 2020, just prior to the impacts of COVID-19 in Australia.

For the purposes of assessing the likely representativeness of the final sample, de-identified data of eligible non-participants were obtained from the VCPR. To compare outcomes of the CP sample with the general population, data were extracted from the Household, Income and Labor Dynamics in Australia (HILDA) 2019 Survey after filtering for the age range of interest (18–25 years) (33, 34). HILDA is a nationally representative survey of Australian households with individual data collected on all household residents over 15 years of age.

### Data Processing and Analysis

All survey responses were either collected directly using REDCap or entered from paper-based questionnaires. All data cleaning and analyses were undertaken using R software version 4.02 (35). Individuals were included in the CP sample if they had returned their survey and provided information for at least one item beyond the demographic characteristics. A comparison of the respondents to invited non-respondents was undertaken using de-identified VCPR data. This comparison was to assess similarity of key CP characteristics between the groups, and to quantify how generalisable the findings would be to the CP population.

Responses to closed-ended questions were summarized using descriptive statistics and described under domains of interest. In the presence of incomplete survey responses, analysis was conducted on observations with complete information only, and frequency of missing data summarized in tabulated results. For the first research question, social outcomes for young adults with CP were reported and compared to a same-aged cohort without CP (via the HILDA dataset restricted to ages 18–25 years). Comparison between the cohorts was made using two approaches. First, responses to common questions within both datasets were summarized and compared descriptively. Second, when appropriate to aid discussion, logistic regression was used to investigate the impact of CP on dichotomised social outcomes of interest, whilst adjusting for potential confounding effects of gender and age. A separate logistic regression model was fitted for each social outcome, with the estimated odds ratio used to quantify the strength of association between exposure (CP vs. no CP) and outcome (e.g., employed vs. unemployed). Odds ratios are reported with their 95% confidence intervals (CI) and probability values to aid in interpretation of the effect size, variability in the observed data, and strength of the evidence, not to make a dichotomized decision of significant/not significant (36, 37). A descriptive comparison was further used to compare the social outcomes of the current CP cohort with that of the 2007 cohort.

For the second research question, responses to questions around potential determinants (individual and environmental factors) of social outcomes were summarized using descriptive statistics within the cohort with CP. To describe the functional impact of CP characteristics on an individual, function was categorized into three levels: high (those classified at Level I or II on GMFCS, MACS, CFCS and with no intellectual impairment or learning disability only), low (those classified at Level IV or V on GMFCS, MACS, CFCS, and severe or moderate intellectual disability) or medium (individuals not in either high or low category). The distribution of functional level was then described (using proportions) to identify the probability of the social outcomes given functional status. Where relevant, and enough responses were obtained, univariate logistic regression was further used to estimate the impact of these factors separately on dichotomized social outcomes for the CP cohort. As before, a separate logistic regression model was fitted for each factor and social outcome of interest. For the third research question, survey responses pertaining to life satisfaction were summarized and described for the young adults with CP.

## Results

### Response Rates and CP Group Characteristics

In total, 110 surveys were returned (16.9% response rate); one duplicate was removed, and 90 surveys had complete information in relevant fields and were included in the analyses. Of these, 61.1% were completed by self-report (*n* = 55), defined as individuals making decisions themselves (regardless of who completed the survey), and 38.9% by proxy (*n* = 35), of which most (*n* = 32) were completed by a parent and *n* = 3 by a carer or other responsible person.

In total, 43.3% (*n* = 39) of individuals were female, and 56.7% male (*n* = 51). The mean age of participants at time of survey completion was 22.4 years (SD 2.2 years, range 18.6 to 25.8 years). Participants included those classified at each level of the GMFCS (22), Manual Ability Classification System [MACS; (23)], Communication Function Classification System [CFCS; (24)], and the Eating and Drinking Ability Classification System [EDACS; (25)] functional classification systems, indicating the sample represented the diversity of CP characteristics (see Table 2). Participants with no intellectual disability made up 45.6% (*n* = 41) of the total, while the proportion of those with moderate or severe intellectual disability was 37.8% (*n* = 34). Comparison of the CP cohort with VCPR non-respondents suggested the distribution of functional abilities was comparable between the two groups, with the age of non-participants being slightly younger (see Table 3). The results pertaining to each research question are reported sequentially within each social outcome domain of interest.

**Table 2:** Characteristics of the CP cohort by survey reporting status.

	**Missing^**a**^ *n* (%)**	**Total CP cohort**	**Self-report group**	**Proxy-report group**
*n*		90	55	35
Topographical distribution—*n* (%)	0 (0)			
Both sides of body		52 (57.8)	28 (50.9)	24 (68.6)
Only on one side of body		29 (32.2)	20 (36.4)	9 (25.7)
Other		9 (10.0)	7 (12.7)	2 (5.7)
Mobility: GMFCS—*n* (%)	0 (0)			
Level I		25 (27.8)	24 (43.6)	1 (2.9)
Level II		27 (30.0)	19 (34.5)	8 (22.9)
Level III		11 (12.2)	4 (7.3)	7 (20.0)
Level IV		10 (11.1)	3 (5.5)	7 (20.0)
Level V		17 (18.9)	5 (9.1)	12 (34.3)
Manual ability: MACS—*n* (%)	0 (0)			
Level I		18 (20.0)	17 (30.9)	1 (2.9)
Level II		34 (37.8)	25 (45.5)	9 (25.7)
Level III		14 (15.6)	8 (14.5)	6 (17.1)
Level IV		9 (10.0)	3 (5.5)	6 (17.1)
Level V		15 (16.7)	2 (3.6)	13 (37.1)
Communication function: CFCS—*n* (%)	0 (0)			
Level I		42 (46.7)	39 (70.9)	3 (8.6)
Level II		17 (18.9)	9 (16.4)	8 (22.9)
Level III		5 (5.6)	2 (3.6)	3 (8.6)
Level IV		15 (16.7)	3 (5.5)	12 (34.3)
Level V		11 (12.2)	2 (3.6)	9 (25.7)
Eating and drinking: EDACS—*n* (%)	0 (0)			
Level I		42 (46.7)	37 (67.3)	5 (14.3)
Level II		24 (26.7)	13 (23.6)	11 (31.4)
Level III		9 (10.0)	4 (7.3)	5 (14.3)
Level IV		8 (8.9)	1 (1.8)	7 (20.0)
Level V		7 (7.8)	0 (0.0)	7 (20.0)
Intellectual ability—*n* (%)	0 (0)			
None (normal or better intelligence)		41 (45.6)	37 (67.3)	4 (11.4)
Learning disability		9 (10.0)	8 (14.5)	1 (2.9)
Mild		6 (6.7)	5 (9.1)	1 (2.9)
Moderate		20 (22.2)	4 (7.3)	16 (45.7)
Severe		14 (15.6)	1 (1.8)	13 (37.1)
Vision difficulty—*n* (%)	4 (4.4)			
No		62 (72.1)	44 (81.5)	18 (51.4)
Yes—some difficulty		16 (18.6)	9 (16.7)	4 (11.4)
Yes—a lot of difficulty		5 (5.8)	1 (1.9)	7 (20.0)
Cannot do at all		3 (3.5)	0 (0.0)	3 (8.6)
Hearing difficulty—*n* (%)	1 (1.1)			
No		78 (87.6)	49 (90.7)	29 (82.9)
Yes—some difficulty		9 (10.1)	4 (7.4)	5 (14.3)
Yes—a lot of difficulty		2 (2.2)	1 (1.9)	1 (2.9)
Cannot do at all		0 (0.0)	0 (0.0)	0 (0.0)
Additional health condition: Yes—*n* (%)	1 (1.1)	35 (39.8)	15 (42.9)	15 (42.9)
PROMIS Physical Health: mean (SD)	5 (5.6)	45.0 (8.5)	47.0 (8.7)	41.8 (7.2)
PROMIS Mental Health: mean (SD)	5 (5.6)	43.1 (9.9)	44.1 (11.3)	41.2 (7.0)
K10 Mental health—*n* (%)	7 (7.8)			
Likely to be well (score <20)		45 (54.2)	22 (41.5)	23 (76.7)
Mild disorder (scores 20–24)		9 (10.8)	7 (13.2)	2 (6.7)
Moderate disorder (scores 25–29)		12 (14.5)	9 (17.0)	3 (10.0)
Severe disorder (scores 30+)		17 (20.5)	15 (28.3)	2 (6.7)
PROMIS Fatigue—*n* (%)	4 (4.4)			
None		10 (11.6)	6 (11.3)	4 (12.1)
Mild		28 (32.6)	19 (35.8)	9 (27.3)
Moderate		37 (43.0)	21 (39.6)	16 (48.5)
Severe		7 (8.1)	6 (11.3)	1 (3.0)
Very severe		4 (4.7)	1 (1.9)	3 (9.1)
PROMIS Pain rating (range 0–10)—mean (SD)	3 (3.3)	2.5 (2.4)	2.7 (2.6)	2.2 (2.1)
Self-care support needs—*n* (%)	1 (1.1)			
Always/sometimes need help and/or supervision		47 (52.8)	15 (27.8)	32 (91.4)
Have difficulty but don't need help and/or supervision		7 (7.9)	6 (11.1)	1 (2.9)
Don't have difficulty but use aids/equipment		5 (5.6)	4 (7.4)	1 (2.9)
Have no difficulty		30 (33.7)	29 (53.7)	1 (2.9)
Domestic care support needs—*n* (%)	1 (1.1)			
Always/sometimes need help and/or supervision		58 (65.2)	23 (42.6)	35 (100)
Have difficulty but don't need help and/or supervision		6 (6.7)	6 (11.1)	0 (0.0)
Don't have difficulty but use aids/equipment		2 (2.2)	2 (3.7)	0 (0.0)
Have no difficulty		23 (25.8)	23 (42.6)	0 (0.0)
General management support need—*n* (%)	2 (2.2)			
Always/sometimes need help and/or supervision		55 (62.5)	20 (37.7)	34 (97.1)
Have difficulty but don't need help and/or supervision		9 (10.2)	8 (15.1)	1 (2.9)
Don't have difficulty but use aids/equipment		3 (3.4)	3 (5.7)	0 (0.0)
Have no difficulty		22 (25.0)	22 (41.5)	0 (0.0)

a *In the presence of missing data, presented percentages are relative to records with complete responses for characteristic of interest*.

**Table 3:** Comparison of the CP cohort with VCPR non-respondents.

	**Missing^**a**^**	**Non-**	**Missing^**a**^**	**Respondents**
	***n* (%)**	**respondents ^**b**^**	***n* (%)**	
*n*		562		87
Sex: Male—*n* (%)	0 (0.0)	337 (60.0)	0 (0.0)	46 (52.9)
Age, years—mean (SD)		20.7 (2.1)		21.2 (1.9)
Motor topography—*n* (%)	3 (0.5)		0 (0.0)	
Hemiplegia/monoplegia		214 (38.3)		27 (31.0)
Diplegia/triplegia		171 (30.6)		26 (29.9)
Quadriplegia		174 (31.1)		34 (39.1)
Mobility: GMFCS—*n* (%)	10 (1.8)		1 (1.1)	
Level I		193 (35.0)		25 (29.1)
Level II		168 (30.4)		26 (30.2)
Level III		57 (10.3)		11 (12.8)
Level IV		67 (12.1)		13 (15.1)
Level V		67 (12.1)		11 (12.8)
Speech—*n* (%)	30 (5.3)		1 (1.1)	
No impairment		211 (39.7)		34 (39.5)
Some impairment		192 (36.1)		29 (33.7)
Non-verbal		129 (24.2)		23 (26.7)
Intellect—*n* (%)	28 (5.0)		2 (2.2)	
Probably no impairment		268 (50.2)		44 (51.8)
Probable impairment		266 (49.8)		41 (48.2)
Vision—*n* (%)	33 (5.9)			2 (2.2)
Not blind		507 (95.8)		81 (95.3)
Blind		22 (4.2)		4 (4.7)
Hearing—*n* (%)	29 (5.2)		1 (1.1)	
Not deaf bilaterally		470 (88.2)		77 (89.5)
Deaf bilaterally		63 (11.8)		9 (10.5)

a *In the presence of missing data, presented percentages are relative to records with complete responses for characteristic of interest.*

b *Presented characteristics were obtained from the VCPR, with small discrepancies between those reported within the surveys for the respondents*.

### Social Outcomes

After restricting to the age range of interest (18 to 25 years), the HILDA cohort included information on 2,375 individuals. In comparison to the CP cohort, there was a smaller proportion of males in the HILDA group (49.1% HILDA compared to 56.7% CP cohort), with the mean age being similar between the cohorts [21.7years (SD: 2.3) HILDA; 22.4years (SD: 2.2) CP cohort]. Key demographic information and social outcomes for both cohorts are presented in Table 4, with specific outcomes discussed in further detail below.

**Table 4:** Comparison of demographic characteristics and social outcomes for CP and HILDA cohorts.

	**Missing^**a**^**	**CP cohort**	**Missing^**a**^**	**HILDA cohort**
	***n* (%)**		***n* (%)**	
*n*		90		2375
**Demographic characteristics**	0 (0.0)		0 (0.0)	
Sex: Male—*n* (%)		51 (56.7)		1165 (49.1)
Age, years—mean (SD)		22.4 (2.2)		21.7 (2.3)
**Highest qual. after school—*****n*** **(%)**	7 (8.4)		0 (0.0)	
University degree		16 (19.3)		365 (15.4)
Associate degree of diploma		4 (4.8)		126 (5.3)
Certificate III or IV		2 (2.4)		434 (18.3)
Other certificate		12 (14.5)		0 (0.0)
Did not complete further education		49 (59.0)		1450 (61.1)
**Employment Status—*****n*** **(%)**	1 (1.1)		0 (0.0)	
Employed		29 (32.6)		1799 (75.8)
Unemployed		16 (18.0)		196 (8.3)
Not in labor force		44 (49.4)		380 (16.0)
**Employment: Psychosocial job quality** ^**b**^				
Scores (0 = strongly disagree, 6 = Strongly agree)—mean (SD)				
Job demands and complexity	1 (3.4)	2.5 (1.3)	282 (15.7)	2.8 (1.1)
Job security	3 (10.3)	3.8 (1.6)	278 (15.5)	3.8 (0.9)
Job control	0 (0.0)	2.7 (2.0)	287 (16.0)	2.7 (1.5)
**Annual income before tax—*****n*** **(%)**	39 (43.3)		0 (0.0)	
<20,000 AUD		32 (62.8)		1118 (47.1)
≥ 20,000 AUD		19 (37.3)		1257 (52.9)
**Marital status—*****n*** **(%)**	2 (2.2)		0 (0.0)	
Married or partnered		3 (3.4)		750 (31.6)
Married		1 (1.1)		125 (5.3)
Defacto		2 (2.3)		625 (26.3)
Single		85 (96.6)		1625 (68.4)
Single, never married		85 (96.6)		1619 (68.2)
Separated		0 (0.0)		5 (0.2)
Divorced		0 (0.0)		1 (0.04)
**Live away from parental home: Yes—*****n*** **(%)**	2 (2.2)	11 (12.5)	0 (0.0)	1230 (51.8)
**Active member of club/association: Yes—*****n*** **(%)**	3 (3.3)	36 (41.4)	338 (14.2)	611 (30.0)
**Frequency of event attendance—*****n*** **(%)**	3 (3.3)		314 (13.2)	
Never		7 (8.0)		192 (9.3)
Rarely		18 (20.7)		613 (29.7)
Occasionally		28 (32.2)		483 (23.4)
Sometimes		15 (17.2)		431 (20.9)
Often		17 (19.5)		254 (12.3)
Very often		2 (2.3)		88 (4.3)
**Life satisfaction overall—mean (SD)**	2 (2.2)	66.3 (23.3)	0 (0.0)	80.3 (13.6)

a *In the presence of missing data, presented summary statistics are calculated for complete responses only.*

b *Data only includes those who were employed (CP n = 29; HILDA n = 1,799); HILDA, Household Income and Labor Dynamics in Australia; qual, qualification*.

#### Educational Achievement

Just over half of the young adults with CP had received their education through mainstream schools (53.4%, 47/88), with 46.6% (41/88) attending a special school. Within the CP cohort, 79.8% of young adults (71/89) reported completing high school (year 12 or equivalent); a higher proportion than the 50% reported in the previous study (2). Despite the high rate of high-school completion, over half of the young adults with CP had not attempted any additional qualifications after school (59.0%, 49/83). Approximately a third of young adults with CP reported they were currently completing formal tertiary study at the time of survey (30.3%, 27/89).

In comparison to the general population, the proportion of young adults obtaining qualifications after high school was similar between cohorts (41.0% in the CP group compared to 38.9% in the general population), with a similar proportion obtaining a University qualification (15.4 and 19.3%, respectively). Within the CP group, the odds of completing post-secondary education increased with better physical health scores (OR = 1.10, 95% CI: 1.04 to 1.17, *p*< *0.01*). Mental health [PROMIS OR = 1.03, 95% CI: 0.99 to 1.08, *p* = *0.19*; K10 OR = 1.05, 95%CI: 0.99 to 0.11, *p* = 0.11)] and pain (OR = 0.98, 95%CI: 0.82 to 1.18, *p* = *0.85)* had little impact on the odds of completing post-secondary education within the CP group. Individuals with higher functional capacity (i.e., classified at Levels I or II on the functional classification systems and/or no intellectual disability) were more likely to obtain a post-secondary qualification (65.5%, 19/29) compared to those with categorized as medium (33.3%, 15/45) or low (0%, 0/9) capacity. Two thirds of individuals reported that their health affected their participation in education (68.9%, 62/90).

Impacts on education are summarized in Table 5. The following environmental factors were reported to somewhat or greatly affect participation in education: lack of access to transport (35.8%), lack of available education close by (30.7%) and lack of family help or assistance (21.5%). In addition, respondents reported that a lack of confidence (48.7%), along with not having required background experience or education were impediments (62.7%).

**Table 5:** Self-reported factors influencing participation.

	**Missing^**a**^ *n* (%)**	**Study CP cohort**
		**(*N* = 90)**
**Impacts on education**		
Lack of access to transport to get to and from school—*n* (%)	9 (10.0)	
Does not affect what I do		52 (64.2)
Somewhat affects what I can do		17 (21.0)
Greatly affects what I can do		12 (14.8)
Lack of availability of schooling/education close to where I live—*n* (%)	12 (13.3)	
Does not affect what I do		54 (69.2)
Somewhat affects what I can do		14 (17.9)
Greatly affects what I can do		10 (12.8)
Lack of family help or assistance—*n* (%)	11 (12.2)	
Does not affect what I do		62 (78.5)
Somewhat affects what I can do		12 (15.2)
Greatly affects what I can do		5 (6.3)
Lack of confidence—*n* (%)	12 (13.3)	
Does not affect what I do		40 (51.3)
Somewhat affects what I can do		27 (34.6)
Greatly affects what I can do		11 (14.1)
Not having the qualifications, experience or skills—*n* (%)	15 (16.7)	
Does not affect what I do		28 (37.3)
Somewhat affects what I can do		21 (28.0)
Greatly affects what I can do		26 (34.7)
Long-term health affect participation in education: Yes—*n* (%)	0 (0.0)	62 (68.9)
**Impacts on employment**		
Lack of access to transport to get to and from work—*n* (%)	19 (21.1)	
Does not affect the work I can do		41 (57.7)
Somewhat affects the work I can do		17 (23.9)
Greatly affects the work I can do		13 (18.3)
Lack of availability close to home—*n* (%)	19 (21.1)	
Does not affect what I do		30 (42.3)
Somewhat affects what I can do		19 (26.8)
Greatly affects what I can do		22 (31.0)
Lack of family help or assistance—*n* (%)	19 (21.1)	
Does not affect what I do		53 (74.6)
Somewhat affects what I can do		13 (18.3)
Greatly affects what I can do		5 (7.0)
Lack of confidence—*n* (%)	19 (21.1)	
Does not affect what I do		35 (49.3)
Somewhat affects what I can do		28 (39.4)
Greatly affects what I can do		8 (11.3)
Not having the qualification, experience or skills—*n* (%)	21 (23.3)	
Does not affect what I do		20 (29.0)
Somewhat affects what I can do		18 (26.1)
Greatly affects what I can do		31 (44.9)
Long-term health affecting employment participation: Yes—*n* (%)	2 (2.2)	64 (72.7)
**Experiences in employment**		
Experienced unfair treatment or discrimination—*n* (%)		
Looking for a job: Yes	22 (24.4)	16 (26.5)
Applying for a job: Yes	23 (25.6)	12 (17.9)
During a job interview: Yes	23 (25.6)	10 (14.9)
**Choice in living arrangements**		
How spend their time—*n* (%)	4 (4.4)	
No choice at all		6 (7.0)
Some choice		26 (30.2)
Complete choice		54 (62.8)
How spend their money—*n* (%)	5 (5.6)	
No choice at all		9 (10.6)
Some choice		30 (35.3)
Complete choice		46 (54.1)
Where they live—*n* (%)	5 (5.6)	
No choice at all		27 (31.8)
Some choice		28 (32.9)
Complete choice		30 (35.3)
Who they live with—*n* (%)	6 (6.7)	
No choice at all		34 (40.5)
Some choice		21 (25.0)
Complete choice		29 (34.5)
Who they spend time with—*n* (%)	5 (5.6)	
No choice at all		12 (14.1)
Some choice		23 (27.1)
Complete choice		50 (58.8)
**Community and social support**		
Social support (RAND, standardized)—mean (SD)		
Emotional/informational support	9 (10.0)	75.9 (25.7)
Tangible support	4 (4.4)	92.1 (14.3)
Affectionate support	4 (4.4)	87.2 (19.5)
Social interaction	9 (10.0)	74.3 (23.7)
Overall support	15 (16.7)	80.5 (17.4)

a *In the presence of missing data, presented summary statistics are calculated for complete responses only*.

#### Employment Status

The rate of employment was much lower for participants with CP than young adults in the general population (32.6% compared to 75.8%), with a substantially higher rate of individuals with CP not being in the labor force (i.e., not working or looking for work: 49.4% compared to 16.0%). After adjusting for age and sex, the odds of employment were substantially lower for individuals with CP compared to those without CP (adjusted OR: 0.14, 95% CI:0.09 to 0.22, *p* < 0.01). For those working, reported job quality in the CP cohort was consistent with that reported in the general population with relatively low levels of adversity (Psychosocial Job Quality (28): job demand and complexity mean = 2.5 SD 1.3; job security mean = 3.6 SD 1.5; and job control mean = 2.7 SD 2.0 (see Table 4). The majority of employed young adults with CP reported working 20 hours or less in the last week across all jobs (57.7%, 15/26), with 46.4% of employed individuals expressing they wanted to work more hours (13/28).

In comparison to the prior study, there has been little change in the proportion of young adults with CP being employed (36.3%, compared with general population of 80.0%) or not being in the labor force (53.5%, compared with general population of 14.6%) (2).

Within the CP cohort, the odds of being employed increased with better physical health scores (OR = 1.06, CI: 1.01 to 1.13, *p* = *0.04*). However, poorer mental health using the K10 measure was also associated with being employed (i.e., increased odds of being employed with higher K10 scores; OR = 1.06, CI: 1.00 to 1.12, *p* = *0.05*); although the PROMIS mental health score indicated little association with employment status. In addition, reported pain had little impact on the odds of employment (OR = 0.99, 95% CI: 0.82 to 1.19, *p* = 0.91). Individuals with higher functional capacity (i.e., classified at Levels I or II on the functional classification systems and/or no intellectual disability) were more likely to be employed (55.9%, 19/29), compared to those categorized as medium (22.2%, 10/45) or low (0%, 0/10) capacity. Respondents commonly reported their long-term health affected their participation in work (72.7%, 64/88).

Other impacts on participation in employment included experiencing unfair treatment or discrimination during job-seeking, lack of transport, lack of available work close by and a lack of family support (see Table 5). Individual factors reported to somewhat or greatly impact employment included lack of confidence (50.7%) and not having appropriate qualifications or experience (71.0%).

#### Financial Status

Young adults with CP reported having a lower personal income than individuals in the general population, with 62.8% (32/51) reporting an annual income of $20,000 Australian dollars or less (before taxation), compared to 47.1% of those without CP. After adjusting for age and sex, there was a strong impact of CP on earning potential, with the odds of earning over $20,000 being lower for individuals with CP compared to those without CP (adjusted OR: 0.38, 95% CI: 0.20 to 0.69, *p* < 0.01). At the time of survey, ~70.6% (60/85) of individuals with CP were receiving money from the NDIS. Many respondents were receiving the Disability Support Pension (68.8%, 55/80), with a smaller proportion receiving the New Start or unemployment benefit (6.2%, 4/65) or other government benefits (22.8%, 13/57).

#### Partnered or Married

Very few young adults with CP were married or partnered at the time of survey (3.4%, 3/88); a proportion substantially lower than adults in the general population (31.6%), and lower than the prior study of young adults with CP (7.5%, compared to 47.9% in the general population at that time (2). In addition, none of the individuals with CP currently surveyed reported having any children (*n* = 88).

#### Living Arrangements

At the time of survey, a small proportion of young adults with CP reported living away from the parental home (12.5%, 11/88) compared to 51.8% of young adults in the general population. In comparison to the prior study, proportions living away from the parental home were lower for both the CP group (20.9%) and the general population (77.9%) (2).

Within the CP cohort, there was little association between the reported physical and mental health (PROMIS physical health OR = 1.01, 95% CI: 0.94 to 1.09, *p* = *0.77*; PROMIS mental health OR = 1.00, 95% CI: 0.94 to 1.07, *p* = *0.99*) and living away from the parental home. The PROMIS mental health result was consistent with the K10 measure of mental health. In addition, reported pain had little impact on the odds of living away from home (OR = 1.05, 95%CI: 0.80 to 1.35, *p* = *0.69*). Those with a higher functional capacity (i.e., classified at Levels I or II on the functional classification systems and/or no intellectual disability) were more likely to be living away from home (18.2%, 6/33) compared to those categorized as medium (8.9%, 4/44) or low (10%, 1/10) functional capacity. When asked about having choice over their living situation, just over one third expressed they had complete choice over where they lived (35.3%) or who they lived with (34.5%; see Table 5).

#### Community Participation

In comparison to their similar aged peers, a higher proportion of young adults with CP were an active member of a club or association (41.4% compared to 30.0%), with a similar distribution of event attendance frequency between the two groups (see Table 4). Within the CP group, participation in community service tended to be low (22.7%, 20/88), with a very low rate of participation in religious services (77.0%, 67/87 never attend). Between half and two-thirds of respondents reported having complete choice over how they spend their time (62.8%), who they spend time with (58.8% complete) and how they spend their money (54.1%).

In relation to factors that may influence participation in community, on average, young adults with CP reported high levels of emotional/informational support, tangible support, affectionate support, and presence of positive social interactions (mean scores range: 74.3 to 92.1), however there was high variability in responses within the group (see Table 5).

#### Life Satisfaction

Young adults had a lower mean life satisfaction score [Personal Well-being Index (30)] than similar aged peers without disability [mean = 66.3 (SD 23.3), compared with mean = 80.3 (SD 13.6) see Table 4]. Within the CP cohort, individuals reported high satisfaction with their standard of living (mean = 81.3, SD 19.8) and safety (mean = 80.3, SD 24.5). However, they reported lower satisfaction in their health (mean = 69.2, SD = 22.9), achieving in life (mean = 62.0, SD 27.9), being part of a community (mean = 66.4 (SD 26.2) and personal relationships (mean = 63.2, SD = 28.2).

## Discussion

### Overview of Findings

The findings of this survey suggest that the social outcomes of young adults with CP remain significantly different than the general Australian population of the same age (18–25 years), with lower proportions obtaining higher education qualifications, gaining employment, being partnered, living away from the parental home and having reasonable finances. The only area in which there was evidence of greater participation in young adults with CP compared to the general population, was in community clubs and associations. Some explanatory variables were related to functional impairment, that is, having higher levels of functional capacity was associated with better outcomes. There is, however, also some evidence that physical health, mental health and presence of pain play a role in relation to participation. Although we did not have enough data for a robust evaluation of the influence of environmental factors on social outcomes, this group of young adults with CP reported impacts on their participation from known factors (38, 39), for example availability of transport and social supports.

One key and positive finding was that the proportion of young adults with CP completing foundational education has improved since the 2007 survey undertaken: from 50 to ~80%. This may relate to shifts in educational policy that has increasingly required inclusion of students with disability (40). Following completion of Year 12, however, the proportion undertaking further education was similar to the proportion in the prior study (2), and given the slightly older aged cohort in the 2007 study (mean age 24 years), this suggests that ongoing educational attainment may also be improving, however without an equivalent aged cohort it is difficult to tell. While impairments may limit capacity for further education for some young people, the respondents in the current study reported several other factors that acted to constrain their educational participation: lack of transport, availability of programs, social supports, confidence and prior experience or qualifications. Most of these factors are modifiable. In addition, the discrepancy between the 45.6% of participants who reported no intellectual disability or learning difficulty, and the 68.9% who reported health impacts on educational participation, requires further investigation regarding the additional health care supports that may be needed.

Employment outcomes for young adults with CP remain poor and do not appear to have changed in the past 13 years. This is consistent with evidence from broader disability research in Australia (41). Although those with higher functional capacity were more likely to obtain employment, some young adults in the work force (employed or looking for work) reported a range of difficulties associated with obtaining work (unfair treatment or discrimination), and a lack of resources and environmental supports affecting what they can do. Positively, when working, these young adults mostly reported psychosocial job quality in relation to job demands, security and control at very similar levels to their similar aged peers. Once again, health status was commonly identified by this group (73%) as impacting on their participation in employment. The suggestion that those who are working have higher levels of pain and mental health concerns, raises questions as to whether these are particularly resilient individuals, or if there is an impact on health from working. The finding that poorer mental health is associated with increased employment among people with CP is, however, hard to explain particularly as other literature shows that employment rates are low in groups with mental health conditions (42). Previous Australian research has shown that people with disability are more likely to have poor quality jobs than their non-disabled peers which has impacts on their mental health (43). However, our sample reported relatively good job quality. Further research exploring this outcome is needed. In addition, not having the appropriate experience or qualification for employment choices was identified as a potential barrier. Consistent with their reports of impacts on their participation in both education and employment, these young adults' satisfaction with “achieving in life” was also lower than data obtained in 2019 from a representative sample of 2000 Australians [mean = 62.0 compared with mean = 73.5; (44)]. These findings reinforce the importance of optimizing participation in education of young people with disability, both during mandated school years and beyond. Educational attainment at the level of the individual's capacity is a crucial resource for living, a finding highlighted in a recent studying examining what adults with CP identify as enablers of their success in adulthood (45).

Most young adults with CP are living in the parental home, compared to their similar-aged peers. Changes over time (i.e., a higher proportion still living with parents) may be explained by the slightly younger age cohort than in the prior study (2); we expect that more young adults will move out of home as they get older. In addition, the age at which young adults leave the parental home in Australia has increased in recent years, from 2001 when 37% of women, and 47% of men aged 18–29 years were living with a parent, up to 54 and 57% respectively, in 2017. Thus, the gap between those with and without CP is closing, because of changes in the general population, driven by economic (e.g., cost of housing, employment concerns for young adults) and social (e.g., increasing involvement in post-school education) factors (46). Most of the young people with CP in this study expressed that they had no, or only some, choice over where they lived or who they lived with—a finding that is at least partly explained by their continuing to live in the parental home. They also had high levels of satisfaction with their standard of living (mean = 81.3 CP cohort), similar to that of other Australians in 2019 (mean = 78.1) (44). Only a very small proportion were partnered or married. Difficulties in establishing social and sexual relationships have been reported previously (47, 48).

Community participation was the one domain in which young people with CP had higher proportions of participation—in clubs and community organizations—than those without CP. Dependent on the type of club, this may or may not provide opportunity to develop friendships and more intimate relationships. In addition, these young adults with CP indicated they were most likely to have “some” or “complete” choice over who they spent time with, how they spent their money and their time. Their reported personal wellbeing varied, however, depending on the aspect considered, with lower levels of satisfaction being expressed, in comparison to other Australians, in personal relationships (mean = 63.2, compared with 79.4), being part of a community (mean = 66.4, compared with 71.2), overall satisfaction (mean = 66.6, compared with 77.6), and health (mean 69.2 compared with 74.5).

### Contribution to Literature/Knowledge

The age of adolescence has recently been proposed to extend well beyond 18 years, suggesting that the time required to achieve the tasks of that developmental period is also extended (49). Findings of this study for those without CP are consistent with that assumption, with high proportions of young adults continuing to live with parents, still undertaking education, and having limited financial resources. Young adults with neurodevelopmental disabilities have been reported to take even longer than their non-disabled peers to achieve developmental outcomes (50), with an implicit (and sometimes explicit) assumption that the reason for that sits solely within the individual—that is, it is impairment related. While some young adults with CP have severe or even profound intellectual and physical impairments, many do not: and social outcomes for all people occur as a result of the transactional exchanges that occur over time between people and context (51).

The environment matters. CP is a complex heterogeneous condition: many individuals with CP will require ongoing supports: indeed, in this study, only 25–30% had no difficulty and did not require aids or equipment to support their independence in self-care, domestic activities or general management. Given that participation is strongly influenced by the person-environment fit (52), the ongoing large discrepancies in participation outcomes between young adults with CP and their peers suggests more needs to be done to build supportive environments and to work toward meeting our obligations in relation to the United Nations Convention on the Rights of Persons with Disability (53).

The substantial increase in the proportion of young people with CP completing foundational education is very positive and provides an important opportunity for young people to be set on a strong path following school leaving. However, attendance at school, while positive, is not enough: the extent to which young people are engaged in learning and provided with the scaffolded experiences that support their incremental skill development is critical. The young adults with CP in this study reported that lack of confidence was a factor in both their educational and employment participation and that they were not as satisfied with their achievements in life as other Australians. Adults with CP have described needing pathways for developing competence, acceptance and support in school life and skills for making friends (45): these early formative experiences, along with an expectation that they will take part in society from parents, teachers and themselves, (45) provide a foundation for adulthood successes, when the needed contextual supports are also in place.

Despite around 70% of participants reporting having individualized NDIS funding, tangible supports, like accessible transport, availability of suitable education or work and social supports, were commonly reported to be a barrier. Prior research reports varied experiences of the NDIS, with some individuals greatly valuing the additional opportunities afforded for autonomy and participation, and others experiencing great difficulty with accessing and using the scheme in ways that meet their preferences (14). There are high hopes for the NDIS to close the participation gap and social outcomes for people with disability but also concerns about implementation, cost and sustainability (54). Currently we need a stronger body of evidence-based approaches and a more sophisticated evaluation of outcomes than is currently available to inform decisions about what works for whom, and when.

Employment outcomes for many young people with neurodevelopmental disabilities have remained poor over a long period, despite evidence being available about what is needed for improvement. Sheppard et al. (41) describe six key principles to supporting the transition from school to employment for those with disability: (1) the expectation that young people can work; (2) collaboration across sectors; (3) participation in meaningful work during the school years; (4) skills development (of all those involved in school transitions); (5) family involvement; and (6) early transition planning.

The relatively high prevalence of mental health concerns in this group, along with presence of pain and fatigue for more than 50% of individuals suggest greater attention is needed to ensure good quality healthcare in early adulthood. In this sample, nearly 40% had yet to transfer from paediatric to adult health care services. The gap in consistent quality healthcare for adults with CP has been recognized, with significant attention in recent years to transition planning (55). Best practice principles for effective healthcare transition are similar to those for transitioning to employment: (1) collaboration across health systems; (2) capacity building in people and communities starting early; (3) supported navigation (that involves family) through the process; (4) accessible information and resources; and (5) education for young people, parents and healthcare professionals (56).

Participation in meaningful life situations is a fundamental right of all people. Young adults with CP may experience a range of impairments of body functions and structures, but if provided with appropriate supports, participation is possible in many aspects of life. There is good evidence that participation outcomes are influenced by interventions delivered in real-world contexts that are tailored to address the individual-context fit to provide the necessary supports to ensure that learning and engagement are effective (57, 58).

### Limitations

This descriptive study used survey methods and did not achieve a strong response rate [110 returned surveys of 649 VCPR known eligible participants (16.9%)]. Of these only 90 were usable. Along with item-level missing data, this meant that some questions had incomplete information and limited the possible analyses and interpretations. Use of population-based comparative data provided evidence that the sample was broadly representative of the population of those with CP living in Victoria. The potential for response bias is acknowledged, however, the direction of any bias is unknown. A larger cohort of participants would have allowed a more detailed analysis of the factors influencing participation in key aspects of life, and potentially allowed subgroup analyses for some outcomes (e.g., determinants of employment). Our categorisation of functional capacity into three levels may be critiqued, however, it did provide a mechanism for considering a constellation of functional capacities given that individuals with CP may experience varied severity of impairment across functional domains. A more detailed questioning of NDIS supports would have been valuable, given just over 70% of participants were receiving some funding. In addition, the standard questions included in this survey about being partnered or married and presence of social and emotional supports, did not capture information about friendships and intimate relationships—areas in which young people with CP and their parents have previously expressed dissatisfaction (7, 45). This survey sought to replicate data that is commonly sought when measuring “outcomes in adulthood” implicitly labeling participation success as having further education and training, being employed, partnered and living out of the parental home. These are inherent cultural biases that can be perpetuated through research, unless the work is grounded in consumer perspectives.

### Implications for Practice, Policy, Research

There is an ongoing need for attention to be given to supporting young people with CP to achieve meaningful participation. Using evidence already available to us (58), stronger pathways to employment (41) and community (38) participation can be built.

The advantage of survey methods is the capacity to reach—potentially—large numbers of participants who are geographically dispersed. Future surveys are likely to be more helpful, however, if there is greater involvement of young adults with CP, their parents and other support people in their development to ensure that their lived experiences assists in designing surveys in which; (1) the most important (or a broader range of) life situations are asked about; (2) significant individual and environmental influences on outcomes are captured; and (3) methods of engaging with individuals are broader (e.g., delivered as an interview) to ensure that those who are able to provide their own, rather than a proxy response, can do so. In addition, surveys that are followed up by more in-depth interviews with those who can provide rich information about specific life situations will provide more nuanced knowledge to inform policy and practice.

## Conclusion

Findings of this study indicate substantial increase in the proportion of young people with CP completing school since the prior study in 2007, but less evidence of improved participation outcomes in employment or living situation. This is over a decade in which substantial disability policy reform has occurred in Australia; but there is still much work to be done. Closing the gap between those with and without CP will require addressing multiple issues at the individual level including reducing pain, improving mental health and building confidence and self-esteem. Environmental and contextual changes are an imperative to achieve the vision of an inclusive society where people with disability have the same opportunity as others to live productive and meaningful lives.

## Data Availability Statement

The datasets presented in this article are not readily available because we did not seek participant consent for re-use of the data. Requests to access the datasets should be directed to christine.imms@unimelb.edu.au.

## Ethics Statement

The studies involving human participants were reviewed and approved by Australian Catholic University Human Research Ethics Committee. The patients/participants provided their written informed consent to participate in this study.

## Author Contributions

CI, DR, and AK conceived and designed the study and obtained the grant funding. CI led the development of the survey, supervised the data collection phase, and drafted the manuscript. DS undertook data cleaning and analyses. All authors contributed to the interpretation of findings, reviewed and contributed to the preparation of the manuscript, and approved the final version.

## Funding

This study received funding from the Disability Research Initiative from the University of Melbourne. The research was undertaken under the auspices of two Australian National Health and Medical Research Council funded Centres for Research Excellence: CRE-Cerebral Palsy (APP1057997) and the Australian Centre for Health, Independence, Economic Participation and Value Enhanced Care for adolescents and young adults with CP (CP-Achieve) (APP1171758). The authors acknowledge the support provided by the Victorian Cerebral Palsy Register, funded by the Royal Children's Hospital Foundation and Victorian Department of Health. The funding agencies were not involved in the conduct of the study or interpretation or reporting of the findings. This study uses unit record data from the Household Income and Labour Dynamics in Australia (HILDA) survey. The HILDA project was initiated and is funded by the Australian Government Department of Social Services and managed by the Melbourne Institute at the University of Melbourne.

## Author Disclaimer

The findings and views reported in this paper based on access to HILDA data are those of the authors and should not be attributed to either the Department of Social Services or the Melbourne Institute.

## Conflict of Interest

The authors declare that the research was conducted in the absence of any commercial or financial relationships that could be construed as a potential conflict of interest.

## Publisher's Note

All claims expressed in this article are solely those of the authors and do not necessarily represent those of their affiliated organizations, or those of the publisher, the editors and the reviewers. Any product that may be evaluated in this article, or claim that may be made by its manufacturer, is not guaranteed or endorsed by the publisher.
